# 3D diffusion‐weighted ^129^Xe MRI for whole lung morphometry

**DOI:** 10.1002/mrm.26960

**Published:** 2017-10-16

**Authors:** Ho‐Fung Chan, Neil J. Stewart, Graham Norquay, Guilhem J. Collier, Jim M. Wild

**Affiliations:** ^1^ POLARIS, Academic Unit of Radiology University of Sheffield Sheffield UK

**Keywords:** hyperpolarized ^129^Xe, lung morphometry, compressed sensing, stretched exponential model, hyperpolarized ^3^He

## Abstract

**Purpose:**

To obtain whole lung morphometry measurements from ^129^Xe in a single breath‐hold with 3D multiple b‐value ^129^Xe diffusion‐weighted MRI (DW‐MRI) with an empirically optimized diffusion time and compressed sensing for scan acceleration.

**Methods:**

Prospective three‐fold undersampled 3D multiple b‐value hyperpolarized ^129^Xe DW‐MRI datasets were acquired, and the diffusion time (Δ) was iterated so as to provide diffusive length scale (Lm_D_) estimates from the stretched exponential model (SEM) that are comparable to those from ^3^He. The empirically optimized ^129^Xe diffusion time was then implemented with a four‐fold undersampling scheme and was prospectively benchmarked against ^3^He measurements in a cohort of five healthy volunteers, six ex‐smokers, and two chronic obstructive pulmonary disease patients using both SEM‐derived Lm_D_ and cylinder model (CM)‐derived mean chord length (Lm).

**Results:**

Good agreement between the mean ^129^Xe and ^3^He Lm_D_ (mean difference, 2.2%) and Lm (mean difference, 1.1%) values was obtained in all subjects at an empirically optimized ^129^Xe Δ = 8.5 ms.

**Conclusion:**

Compressed sensing has facilitated single‐breath 3D multiple b‐value ^129^Xe DW‐MRI acquisitions, and results at ^129^Xe Δ = 8.5 ms indicate that ^129^Xe provides a viable alternative to ^3^He for whole lung morphometry mapping with either the SEM or CM. Magn Reson Med 79:2986–2995, 2018. © 2017 The Authors Magnetic Resonance in Medicine published by Wiley Periodicals, Inc. on behalf of International Society for Magnetic Resonance in Medicine. This is an open access article under the terms of the Creative Commons Attribution License, which permits use, distribution and reproduction in any medium, provided the original work is properly cited.

## INTRODUCTION

The apparent diffusion coefficient (ADC) calculated from hyperpolarized ^3^He diffusion‐weighted MRI (DW‐MRI) has been shown to be sensitive to changes in lung microstructure 1, 2. The non‐Gaussian diffusion behavior of the gas in the lungs results in a non‐monoexponential signal attenuation with increasing b‐value 3. The signal decay is determined by experimental and physiological factors including gas diffusivity, diffusion gradient strengths and timings, and the complexity of alveolar microstructure, which together influence the measurement of ADC 4, 5. Theoretical diffusion models, such as the cylinder model (CM) 6, 7, stretched exponential model (SEM) 8, and q‐space analysis 9, have been proposed to model this non‐Gaussian diffusion behavior and derive estimates of alveolar length scales (i.e., morphometry) from multiple b‐value DW‐MRI acquisitions. Compressed sensing (CS) has enabled multiple b‐value ^3^He DW‐MRI for 3D whole lung morphometry mapping in a single breath‐hold 10 for quantitative regional assessment of lung microstructure.

With the limited availability of ^3^He gas 11, ^129^Xe provides a more cost‐effective alternative for pulmonary MRI, and with advancements in polarization levels 12, 13, recent studies have shown that comparable ventilation and microstructural information can be obtained using both nuclei 14, 15, 16, 17. DW‐MRI with ^129^Xe is, however, inherently more challenging due to the lower diffusivity and gyromagnetic ratio of ^129^Xe compared with ^3^He, resulting in longer diffusion gradient times, longer sequence echo time (TE) and repetition time (TR), and lower image SNR. Despite these challenges, theoretical models have been proposed for interpreting the ^129^Xe DW‐MRI signal from multiple b‐value acquisitions 18, and estimates of alveolar length scales have been derived from healthy subjects and chronic obstructive pulmonary disease (COPD) patients 19, 20, 21. However, the multiple b‐value interleaves in previous studies were acquired using noncontiguous, relatively thick 2D slices without whole lung coverage—and in some cases in separate breath‐holds—due to the associated long scan times. Furthermore, to our knowledge, no direct comparison of alveolar length scales derived from application of theoretical diffusion models of ^3^He and ^129^Xe in vivo have yet been presented.

In this study, compressed sensing acceleration methods developed for ^3^He 10 were adapted for 3D multiple b‐value ^129^Xe DW‐MRI in a single breath‐hold, and 3D morphometric maps of mean diffusive length scale (Lm_D_) were generated using the SEM. Results were compared against equivalent 3D ^3^He Lm_D_ morphometric maps acquired with CS, and an optimal ^129^Xe diffusion time of Δ = 8.5 ms was derived empirically. Prospective acquisitions with the optimal ^129^Xe diffusion time were then benchmarked in healthy volunteers, ex‐smokers, and COPD patients with both SEM‐derived Lm_D_ and CM‐derived mean chord length (Lm) measurements.

## THEORY

### The Stretched Exponential Model

The non‐Gaussian signal decay from an imaging voxel can be modeled as the superposition of signals with different apparent diffusivities ( D):

(1) $$\frac{S_{b}}{S_{0}}=\int_{0}^{D_{0}}p\left(D\right)e^{-bD}dD$$

where $S_{0}$ is the signal when b=0, $S_{b}$ is the signal corresponding to a non‐zero b‐value, D are all possible apparent diffusivities between 0 and $D_{0}$ (the free diffusion coefficient of ^3^He or ^129^Xe in air/N_2_), and p(D) is the probability distribution associated with the apparent diffusivities. The non‐Gaussian HP gas diffusion signal decay in the lungs can be well described by an SEM fit (Equation (2)) 22.

(2) $$\frac{S_{b}}{S_{0}}=e^{{\left[-b\;DDC\right]}^{\alpha }}$$

With ^3^He DW‐MRI, the SEM‐derived parameters of distributed diffusivity coefficient (DDC) and heterogeneity index (α) have been shown to be sensitive to changes in lung microstructure and are valid over a range of experimental conditions. DDC is dependent on diffusion time, while α has been demonstrated to be insensitive to lung inflation and experimental diffusion time 23. A numerical expression for p(D) can be estimated from the SEM‐derived parameters using the approach developed by Berberan‐Santos et al. 24:

(3) $$p\left(D\right)=\tau_{0}\frac{B}{D\tau_{0}{}^{\left(1-\alpha /2\right)/\left(1-\alpha \right)}}\cdot \text{exp}\left[-\frac{\left(1-\alpha \right)\alpha^{\alpha /\left(1-\alpha \right)}}{D\tau_{0}{}^{\alpha /\left(1-\alpha \right)}}\right]\cdot f\left(D\right),$$

where $\tau_{0}$ is 1/DDC, and f(D) is defined by

(4) $$f\left(D\right)=\{\begin{array}{l} 1/[1+C{\left(D\tau_{0}\right)}^{\delta }],\;\delta =\alpha \left(0.5-\alpha \right)/\left(1-\alpha \right),\;\alpha \leq 0.5,\\ \left[1+C{\left(D\tau_{0}\right)}^{\delta }\right],\;\delta =\alpha \left(\alpha -0.5\right)/\left(1-\alpha \right),\;\alpha >0.5, \end{array}.$$

The parameters B and C are functions related to α, and parameters at specific α values can be found in Table 1 of Berberan‐Santos et al. 24. Interpolation can be used to derive the corresponding parameters B and C for other α values. The expression for p(D) can subsequently be related to a distribution of diffusion length scales $p\left(L_{D}\right)$ associated with the different apparent diffusivities through the 1D diffusion equation L_D_ *=* (2*D*Δ)^½^ (i.e., root mean squared displacements, where Δ is the diffusion time). The $p\left(L_{D}\right)$ distributions should then represent the distribution of microscopic dimensions of the airways (i.e., the diffusion‐restricting boundaries) contained within a given voxel. These distributions can then be used to calculate the mean diffusion length scale (Lm_D_) as a quantitative estimate of the mean acinar airway dimensions within a given voxel. The Lm_D_ metric should therefore be analogous to the calculation of mean linear intercept length (L_x_) from histology.

**Table 1:** Summary of Subject Demographics and Pulmonary Function Test Data

Subjects	Age	Sex	FEV_1_ (% pred)	FEV_1_/FVC (%)	TLC (% pred)	RV (% pred)	T_LCO_ (% pred)	Smoking Pack Years
Healthy volunteers								
HV1	26	Male	102.9	81.2	105.6	107.0	—	—
HV2	31	Male	102.0	82.8	100.3	85.0	—	—
HV3	34	Male	77.0	88.0	91.7	107.2	—	—
HV4	31	Male	105.0	87.0	91.6	70.7	—	—
HV5	33	Male	85.1	76.0	84.0	74.1	—	—
Ex‐smokers								
ES1	47	Female	86.7	68.8	108.4	105.3	93.0	30.0
ES2	51	Male	95.2	69.2	106.7	100.9	97.2	30.0
ES3	53	Female	90.1	59.7	130.0	139.2	99.2	4.1
ES4	55	Male	107.7	67.5	132.0	127.0	86.5	10.0
ES5	52	Female	90.9	71.0	101.9	106.6	89.3	25.0
ES6	50	Male	111.6	96.1	109.0	89.4	98.4	22.5
COPD patients								
COPD1	62	Female	39.6	36.5	—	—	37.4	—
COPD2	64	Female	69.7	50.0	—	—	61.0	—

This method of calculating Lm_D_ differs from the method used to derive mean chord length (Lm) with the CM. In the CM, the underlying assumptions are that the acinar airways are considered cylindrical objects and thus the HP gas diffusion signal can be described by two anisotropic diffusion coefficients, longitudinal (D_L_) and transverse (D_T_). Phenomenological expressions were empirically optimized from Monte Carlo simulations to relate D_L_ and D_T_ to the cylindrical lung airway parameters, outer airway radii (R) and alveolar sleeve depth (h) 6, 25. Lm is subsequently derived from the alveoli surface area and volume based upon the geometrical parameters of R and h 7.

## METHODS

All in vivo MRI experiments were performed under the approval of the UK National Research Ethics Committee and the local National Health Service research office. All CS simulations and lung morphometry calculations were implemented in‐house using MATLAB (MathWorks, Natick, Massachusetts, USA) software. The signal‐to‐noise ratio (SNR) for each dataset was computed in the magnitude images (b = 0) by dividing the mean signal of the entire segmented lung region by a region of background noise corrected for Rician distribution bias. It should be noted that SNR calculated from CS images present a biased measure of SNR, due to the denoising process associated with CS reconstruction.

### 3D Multiple b‐Value ^129^Xe DW‐MRI with CS

A fully sampled 3D ^129^Xe DW‐MRI dataset was acquired from a healthy male volunteer (HV1) on a 1.5 T (GE HDx) MR scanner using a flexible quadrature transmit/receive vest coil (Clinical MR Solutions, Brookfield, Wisconsin, USA) which was tuned to the Larmor frequency of ^129^Xe at 1.5 T (17.66 MHz). All lung imaging was performed at a lung volume of functional residual capacity plus 1L following inhalation of a dose of 800 mL enriched Xe [86% ^129^Xe, ∼30% polarization 12, 13] mixed with 200 mL of N_2_. Image acquisition parameters were: 3D spoiled gradient echo sequence; 2 × interleaves (b = 0, 12 s/cm^2^); elliptical‐centric phase encoding; in‐plane resolution = 64 × 52 (6.25 mm pixel dimension); 18 effective coronal slices (15 mm slice thickness); field of view = 40 × 32.5 × 27 cm^3^; TE/TR = 11.2/14.4 ms; diffusion time (Δ) = 5 ms (diffusion gradient strength = 22.7 mT/m, ramp time = 0.3 ms, plateau time = 3 ms, gap between lobes = 1.4 ms); flip angle = 2.2°; and bandwidth = ±6.97 KHz.

^129^Xe Δ = 5 ms was first chosen as it corresponds to the diffusion time originally proposed for ^129^Xe lung morphometry with the CM 18. This time was derived theoretically such that acinar airway geometrical parameters from the CM for ^129^Xe would be the same as those obtained with ^3^He 18, and these values have been subsequently used in 2D ^129^Xe DW‐MRI experimental studies 20, 21. Retrospective CS simulations of the fully sampled dataset with acceleration factors (AF) between 2 and 5 were implemented using the methodology described previously for ^3^He 10. The Wilcoxon signed‐rank test was employed to assess differences in fully sampled and retrospectively reconstructed ADC maps for each AF on a pixel‐by‐pixel basis.

The optimum k‐space sampling pattern for three‐fold undersampling was chosen based on the simulation results and was used for prospective acquisition of 3D ^129^Xe multiple b‐value DW‐MRI data from four healthy volunteers (HV1, HV2, HV3, HV4). Prospective data were acquired with an inhaled gas mixture of 750 mL ^129^Xe and 250 mL nitrogen, with imaging parameters as for the fully sampled acquisition other than the following: four interleaves (b = 0, 12, 20, 30 s/cm^2^); TE/TR = 11.7/15.0 ms; Δ = 5 ms (maximum diffusion gradient strength = 31.9 mT/m, ramp time = 0.3 ms, plateau time = 3.5 ms, gap = 0.9 ms); and flip angle = 2.7°. The AF of 3 reduces the scan time from 57 to 19 s. ^129^Xe Lm_D_ maps were calculated using the SEM, and results were compared with Lm_D_ maps derived from the same volunteers' lungs using ^3^He DW‐MRI as described by Chan et al. 10. ^3^He Lm_D_ at ^3^He Δ = 1.6 ms was chosen for comparison because healthy and COPD Lm_D_ values derived at this diffusion time have been demonstrated to match histologically derived healthy and COPD mean linear intercept values 26.

### Empirical Determination of Optimal ^129^Xe Diffusion Time

With the aim of obtaining the best agreement between ^129^Xe and ^3^He lung morphometry results [rather than simply using the ^129^Xe Δ = 5 ms proposed by Sukstanskii and Yablonskiy 18], HV1 was imaged at additional diffusion times (Δ = 5, 7, 8, and 10 ms). ^129^Xe Δ = 10 ms was chosen as it corresponds to the same 1D characteristic free diffusion length ( $\sqrt{2D_{0}\Delta }$ ∼530 µm) as experienced in the benchmark ^3^He experiment (assuming $D_{0}^{\text{Xe}–\text{air}}=\;0.14{\;\text{cm}}^{2}/{s,\;D}_{0}^{\text{He}–\text{air}}=\;0.88{\;\text{cm}}^{2}/s$, and Δ^He^ = 1.6 ms). Each additional scan was acquired with the same gas mixture and b‐values as the previous prospective CS acquisitions at ^129^Xe Δ = 5 ms, and Lm_D_ maps were calculated from each dataset.

### Benchmarking of Empirically Optimized ^129^Xe Diffusion Time

The empirically optimized diffusion time (^129^Xe Δ = 8.5 ms [see Results]) was then benchmarked against ^3^He equivalent measurements for lung morphometry mapping over different ranges of acinar length scales that are experienced with smoking‐related emphysema. Five healthy volunteers (age, 31.0 ± 3.1 years), six ex‐smokers (age, 51.3 ± 2.7 years), and two COPD patients (age, 63.0 ± 1.4 years, GOLD II‐IV) were recruited for this preliminary study. Subject demographics and pulmonary function test (PFT) data for each subject are summarized in Table 1.

Each subject was imaged with 3D multiple b‐value ^129^Xe DW‐MRI, using 750 mL of inhaled ^129^Xe and the following imaging parameters: TE/TR = 14.0/17.3 ms; maximum DW gradient strength = 32.6 mT/m; Δ = 8.5 ms; ramp time = 0.3 ms; plateau time = 2.3 ms; gap = 5.6 ms; and flip angle = 3.1°. Using ^129^Xe Δ = 8.5 ms, the duration of three‐fold undersampled CS scans was increased by 3 s due to the increased diffusion time. Therefore, four‐fold undersampling (AF = 4) was now implemented in the subsequent prospective CS acquisitions to further reduce the breath‐hold to 16 s, similar to the 15 s acquisition for ^3^He 10, and to demonstrate the clinical viability of this sequence. 3D ^3^He DW‐MRI was acquired in same‐day scan sessions for all subjects (except for HV1–HV3, for whom ^3^He data were acquired approximately 1 year previously), with experimental parameters described previously 10. ^129^Xe and ^3^He Lm_D_ maps were derived and compared in each subject.

Finally, the applicability of ^129^Xe Δ = 8.5 ms to CM derivations of lung morphometry parameters was assessed. The ^129^Xe‐based CM phenomenological expressions are optimized for ^129^Xe Δ = 5 ms; however, if the same theoretical free diffusion length is probed with both nuclei (i.e., Δ_He_ = 1.6 ms and Δ_Xe_ = 10 ms), the original ^3^He‐based phenomenological expressions should in theory be applicable for derivation of ^129^Xe lung morphometry parameters 18. Initial CM analysis of ^129^Xe DW‐MRI data in healthy subjects at ^129^Xe Δ = 8.5 ms and ^129^Xe Δ = 10 ms, suggested that, as with the SEM, more consistent ^129^Xe lung morphometry results were obtained with ^129^Xe Δ = 8.5 ms (see Discussion). The 3D multiple b‐value ^129^Xe DW‐MRI data at ^129^Xe Δ = 8.5 ms was therefore analyzed using the ^3^He‐based CM phenomenological expressions 7, and the ^129^Xe mean chord length (Lm) was hence derived and compared with ^3^He‐derived Lm for each subject in the preliminary study.

## RESULTS

### 3D Multiple b‐Value ^129^Xe DW‐MRI with CS

**Figure 1:**
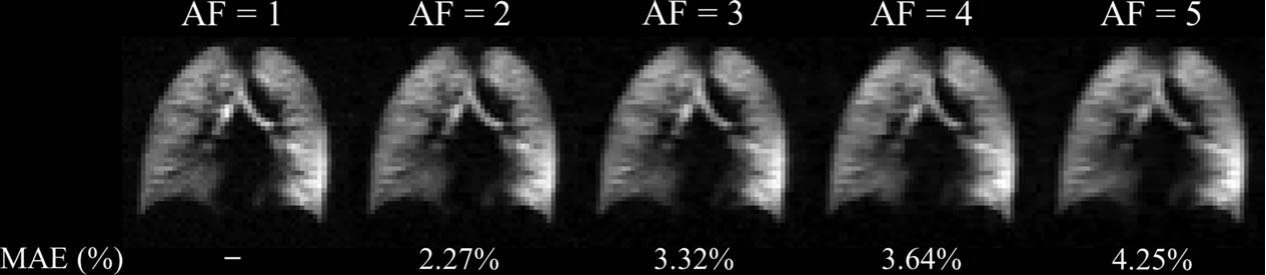
CS simulation results for 3D ^129^Xe DW‐MRI. Reconstructed magnitude image (b = 0) for each AF, with corresponding MAE values (AF = 1; fully sampled dataset [SNR = 25]).

**Figure 2:**
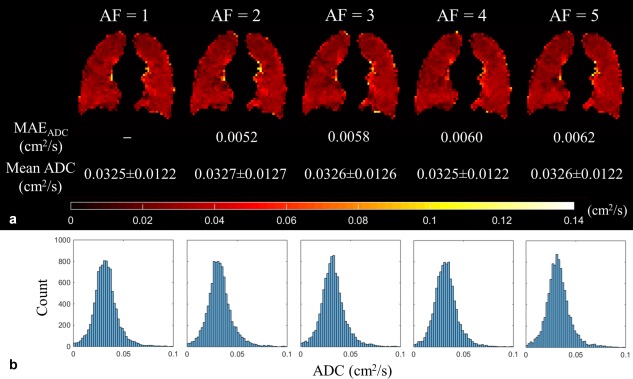
ADC results for 3D ^129^Xe DW‐MRI CS simulations. (**a**) Single‐slice ADC maps with the MAE_ADC_, and mean global ADC values for each AF. (**b**) Corresponding whole lung ADC histograms for each AF.

The SNR of the fully sampled ^129^Xe DW‐MRI dataset was 25. Optimal k‐space undersampling patterns for different AFs were determined through CS simulations. Retrospectively reconstructed datasets from each optimal undersampling pattern showed a small increase in mean absolute error (MAE) of normalized signal intensity value for the b = 0 data (from 2.27% at AF = 2 to 4.25% at AF = 5), indicating a good preservation of image details with increased AF (Fig. 1). Whole lung mean ADC histograms and single slice ADC maps generated from the reconstructed CS datasets also demonstrated a good preservation of quantitative information and low MAE_ADC_ (Fig. 2). Wilcoxon signed‐rank tests for each AF found no significant differences (*P* > 0.05) between CS‐reconstructed and fully sampled ADC maps on a pixel‐by‐pixel basis, confirming preservation of quantitative information and indicating that CS is suitable for 3D ^129^Xe multiple b‐value DW‐MRI.

**Figure 3:**
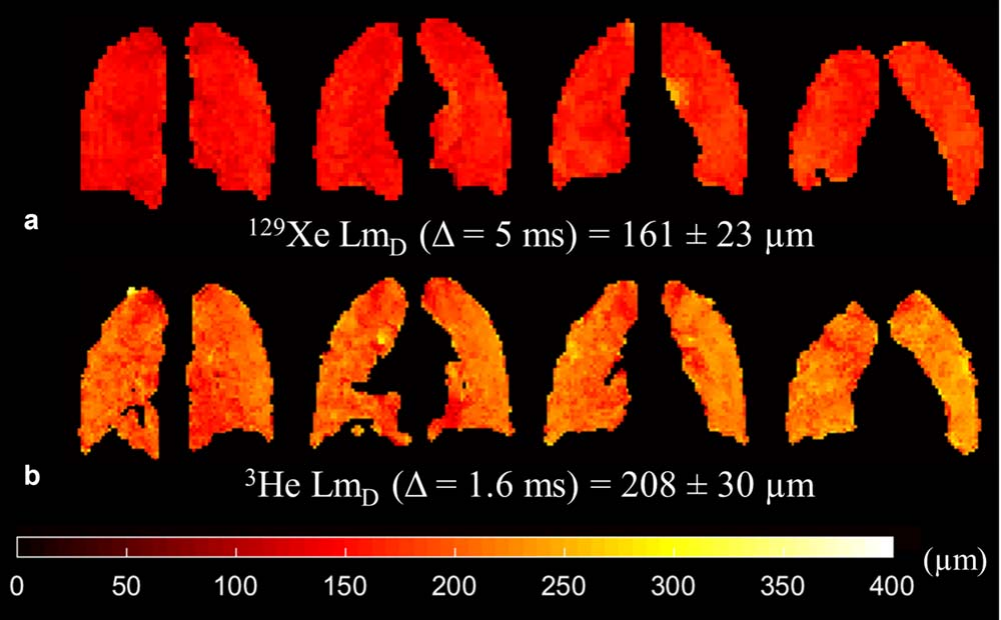
Prospective CS results for a healthy volunteer (HV1) (SNR = 30). (**a**) Example ^129^Xe Lm_D_ maps derived from 3D multiple b‐value ^129^Xe DW‐MRI. (**b**) Example ^3^He Lm_D_ maps in comparative slices demonstrate the mismatch in Lm_D_ values between the two nuclei.

Prospective 3D ^129^Xe multiple b‐value DW‐MRI was acquired in four healthy volunteers with AF = 3 and ^129^Xe Δ = 5 ms, and resulting ADC and Lm_D_ maps were compared with previously calculated lung microstructural maps acquired using 3D ^3^He multiple b‐value DW‐MRI. Mean SNR for the four prospective ^129^Xe datasets was 40. The prospective CS whole lung mean ^129^Xe ADC value for volunteer HV1 (0.0329 cm^2^/s) was very similar (+1.2% difference) to the fully sampled mean ADC value (0.0325 cm^2^/s) that was obtained for CS simulations. Example ^129^Xe and ^3^He Lm_D_ maps from the comparative slices in HV1 are shown in Figure 3 and a summary of mean ADC and Lm_D_ values for each volunteer is provided in Table 2. At ^129^Xe Δ = 5 ms, mean ^129^Xe Lm_D_ values for all subjects were ∼50 µm smaller than the corresponding mean ^3^He values.

**Table 2:** Summary of Whole Lung Mean ADC and Lm_D_ Values for Four Healthy Volunteers Derived from Prospective 3D Multiple b‐Value ^129^Xe and ^3^He DW‐MRI With CS

Subjects	^129^Xe ADC (cm^2^/s) (Δ = 5 ms)	^129^Xe Lm_D_ (µm) (Δ = 5 ms)	^3^He ADC (cm^2^/s) (Δ = 1.6 ms)	^3^He Lm_D_ (µm) (Δ = 1.6 ms)
HV1	0.033 ± 0.012	161 ± 23	0.182 ± 0.085	208 ± 30
HV2	0.039 ± 0.012	176 ± 20	0.196 ± 0.077	223 ± 24
HV3	0.030 ± 0.011	157 ± 19	0.166 ± 0.068	205 ± 23
HV4	0.030 ± 0.011	156 ± 18	0.169 ± 0.065	210 ± 20

### Empirical Determination of Optimal ^129^Xe Diffusion Time

**Figure 4:**
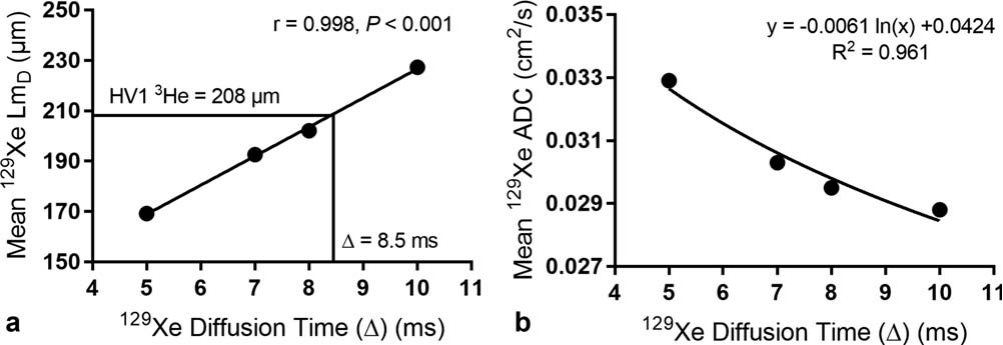
Global mean ^129^Xe Lm_D_ and ADC results at different ^129^Xe diffusion times for one healthy volunteer. (**a**) A strong linear dependence in ^129^Xe diffusion time and mean ^129^Xe Lm_D_ value was observed. At ^129^Xe Δ=8.5 ms, the ^129^Xe Lm_D_ matches the volunteer's corresponding ^3^He Lm_D_ value. (**b**) Mean ^129^Xe ADC decreases with increasing diffusion time in a nonlinear logarithmic relationship.

A strong positive linear correlation (*r* = 0.998, *P* < 0.001) was established between ^129^Xe Lm_D_ and diffusion times, and at Δ = 8.5 ms the ^129^Xe Lm_D_ value best matched the volunteer's ^3^He Lm_D_ value (Fig. 4a). In contrast to Lm_D_, mean ^129^Xe ADC decreased with increasing diffusion time; a 12.5% decrease in mean ^129^Xe ADC was observed from Δ = 5 ms to 10 ms. The relationship between ^129^Xe ADC and diffusion time was nonlinear, however, and best fitted a logarithmic function (R^2^ = 0.961) (Fig. 4b).

### Benchmarking of Empirically Optimized ^129^Xe Diffusion Time

**Figure 5:**
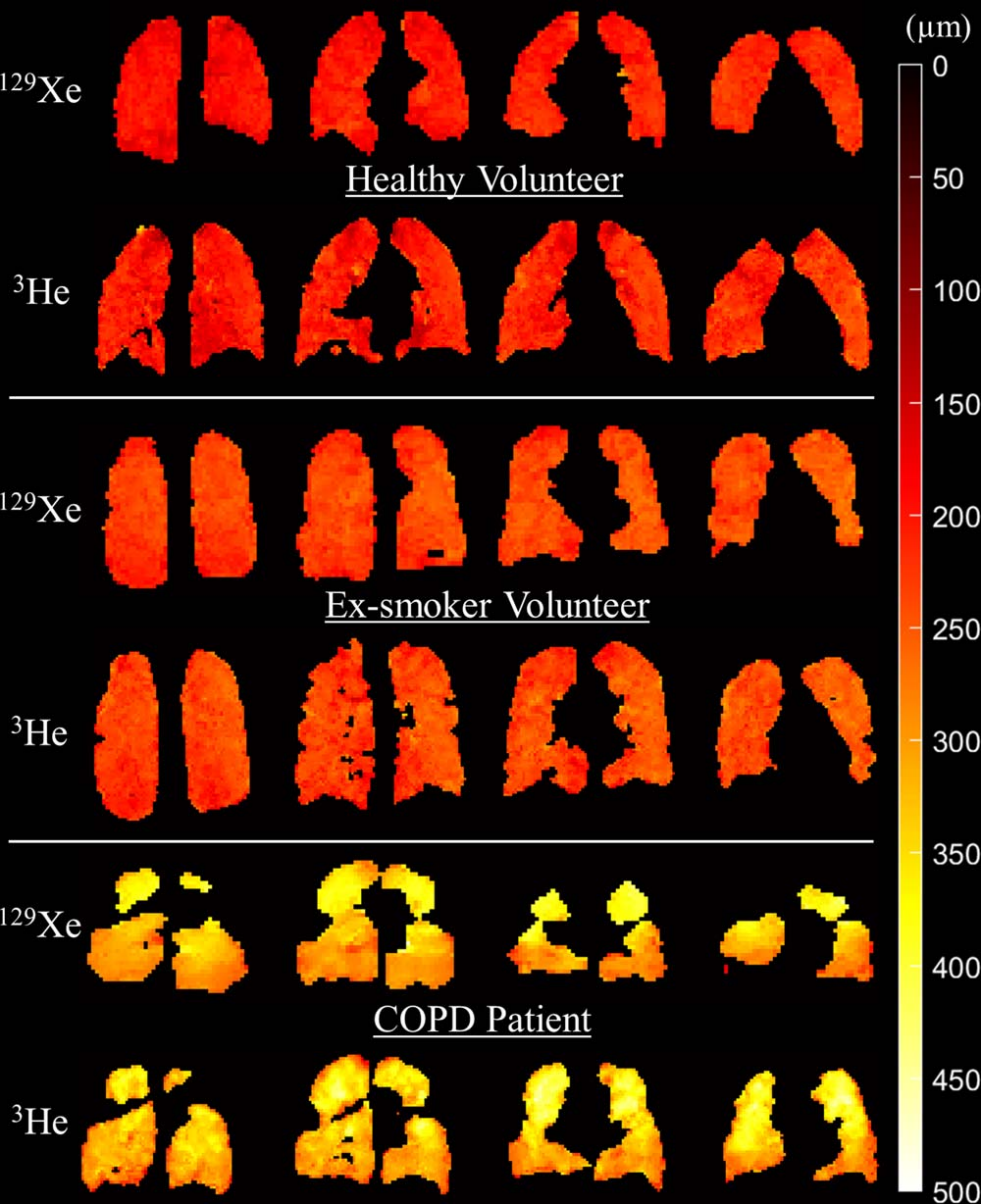
Comparison of ^129^Xe and ^3^He example slice Lm_D_ maps for a representative healthy, ex‐smoker, and COPD subject. ^129^Xe Lm_D_ maps derived using 3D multiple b‐value ^129^Xe DW‐MRI at an empirically optimized diffusion time Δ = 8.5 ms demonstrate good agreement with ^3^He Lm_D_ maps. 3D ^129^Xe DW‐MRI mean SNR was 37, 44, 80 for the representative healthy volunteer, ex‐smoker, and COPD patient, respectively.

**Figure 6:**
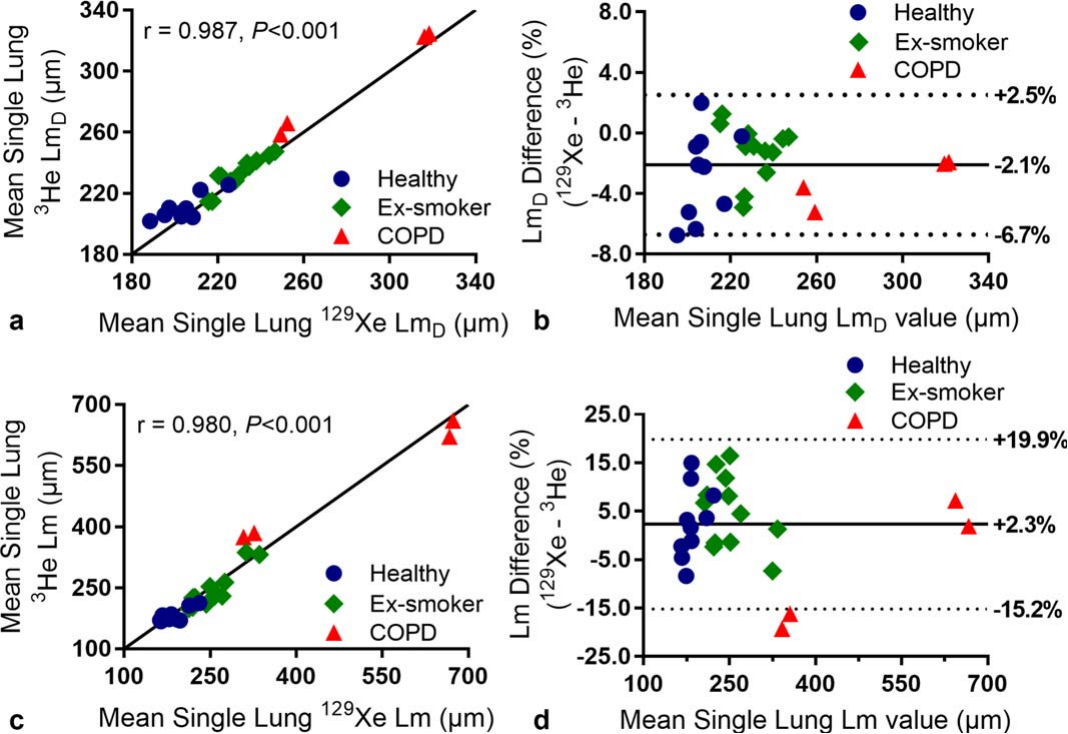
(**a**) Comparison of ^129^Xe and ^3^He mean single (left and right) lung Lm_D_ values for all subjects. The solid line represents the line of equality. (**b**) Bland‐Altman analysis of mean single lung Lm_D_ values. The percentage difference (^129^Xe – ^3^He) between the two nuclei is plotted against the mean single lung Lm_D_ value of the two nuclei for all subjects. The solid line represents the mean percentage difference, and the two dotted lines indicate the 95% (±1.96 SD) difference range. (**c**) Comparison of ^129^Xe and ^3^He mean single lung Lm values derived from the cylinder model for all subjects. Both ^129^Xe and ^3^He data were analyzed with the ^3^He‐based cylinder model. The solid line represents the line of equality. (**d**) Corresponding Bland‐Altman analysis of mean single lung Lm values.

The mean ^3^He and ^129^Xe SNR of the b = 0 image for all preliminary study subjects was 32 and 65, respectively. A summary of ^129^Xe Lm_D_ and corresponding ^3^He Lm_D_ values are shown in Table 3. An improved matching of mean ^129^Xe and ^3^He Lm_D_ was obtained with the empirically optimized diffusion time, and this is visible in example Lm_D_ maps from three representative subjects (Fig. 5). A difference in Lm_D_ of less than 7% was observed in all subjects, with a mean difference (^129^Xe − ^3^He) in all subjects of −2.2%. Figure 6a shows a very strong correlation (*r* = 0.987, *P* < 0.001) between individual lung ^3^He and ^129^Xe mean Lm_D_ values in all subjects. Lm_D_ values fall around the line of equality, and this good agreement was confirmed by Bland‐Altman analysis (Fig. 6b) of individual lung Lm_D_ values, where a mean bias of −2.1% (−4.8 µm) for ^129^Xe mean Lm_D_ with a 95% confidence interval of −6.7% to 2.5% (−14.8 to 5.2 µm) was observed.

**Table 3:** Summary of ^129^Xe Whole Lung SEM‐Derived Lm_D_ and CM‐Derived Lm Values for Healthy Volunteers, Ex‐smokers, and COPD Patients Acquired With AF = 4 and ^129^Xe Δ = 8.5 ms and Their Corresponding ^3^He Mean Lung Morphometry Values (AF = 3, ^3^He Δ = 1.6 ms)

Subjects	Stretched Exponential Model			Cylinder Model (^3^He‐based)		
	^129^Xe Lm_D_ (µm)	^3^He Lm_D_ (µm)	Lm_D_ Difference (%)	^129^Xe Lm (µm)	^3^He Lm (µm)	Lm Difference (%)
Healthy volunteers						
HV1	205	208	−1.4	183	183	0.0
HV2	218	224	−2.7	222	210	+5.6
HV3	206	205	+0.5	196	171	+12.5
HV4	200	210	−4.8	173	178	−3.1
HV5	192	205	−6.3	164	170	−3.6
Mean HV	**204**	**210**	−**2.9**	**188**	**182**	**+2.3**
Ex‐smokers						
ES1	232	234	−0.9	259	222	+14.3
ES2	230	234	−1.7	254	240	+5.3
ES3	234	236	−0.8	266	250	+6.0
ES4	245	246	−0.4	326	335	−2.7
ES5	221	231	−4.3	222	226	−2.1
ES6	217	215	+0.9	217	201	+7.2
Mean ES	**230**	**233**	−**1.2**	**257**	**246**	**+4.7**
COPD patients						
COPD1	317	323	−1.9	639	671	−5.0
COPD2	251	263	−4.6	318	381	−19.8
Mean COPD	**284**	**293**	−**3.2**	**478**	**526**	−**12.4**
Overall mean	**—**	**—**	−**2.2**	**—**	**—**	**+1.1**

The mean difference in ^129^Xe and ^3^He CM Lm values was +1.1% (Table 3), demonstrating a similar level of agreement in CM‐derived Lm at ^129^Xe Δ = 8.5 ms as SEM‐derived Lm_D_. ^3^He and ^129^Xe CM single lung Lm values were also strongly correlated (*r* = 0.980, *P* < 0.001) (Fig. 6c), and Bland‐Altman analysis of mean single lung Lm values indicates a mean bias of +2.3% in ^129^Xe Lm values with a 95% confidence interval of −15.2% to 19.9% (Fig. 6d).

## DISCUSSION

### 3D Multiple b‐Value ^129^Xe DW‐MRI with CS

CS has enabled the acquisition of 3D multiple b‐value ^129^Xe DW‐MRI in a single breath‐hold for the generation of whole lung maps of alveolar diffusion length scale with a voxel size of 6.25 × 6.25 × 15 mm^3^. Retrospectively undersampled ^129^Xe datasets demonstrated good preservation of image details and microstructural information with increased undersampling. MAE and MAE_ADC_ values from ^129^Xe CS simulations were similar to those reported with ^3^He 10. The presence of image blurring in the fully sampled ^129^Xe images is likely the result of elliptical‐centric phase encode ordering used with ^129^Xe in contrast to sequential encoding used previously with ^3^He. Elliptical‐centric phase encoding maximizes SNR at the consequence of increased image blurring with a RF depolarization *k*‐space filter that originates from the center of k‐space 27. The full width at half maximum values of retrospectively undersampled ^129^Xe ADC histograms decreased with AF; this trend matches the results of ^3^He CS simulations 10 and demonstrates decreased spatial heterogeneity associated with the de‐noising reconstruction process of CS. However, this loss of spatial heterogeneity did not result in a statistically significant difference between fully sampled ADC and undersampled CS ADC maps.

Prospective three‐fold undersampled 3D multiple b‐value ^129^Xe DW‐MRI was acquired in four healthy volunteers at Δ = 5 ms. The difference of + 1.2% between CS (0.0329 cm^2^/s) and fully sampled mean ^129^Xe ADC (0.0325 cm^2^/s) for one volunteer (HV1) was similar to the small differences we reported previously between fully sampled and CS undersampled 2D and 3D ^3^He ADC values 10, 28. The whole lung mean ^129^Xe ADC value for all four healthy volunteers (∼0.033 cm^2^/s) was also consistent with previously reported healthy subject ADC values, with b = 12 s/cm^2^ at 1.5 T 29. The observed mean Lm_D_ mismatch of approximately 50 µm between ^3^He and ^129^Xe suggests that the ^129^Xe diffusion time of Δ = 5 ms, previously proposed for in vivo lung morphometry with the CM 18, is not applicable for ^129^Xe lung diffusion length scale measurements derived from the SEM.

### Empirical Determination of Optimal ^129^Xe Diffusion Time

**Figure 7:**
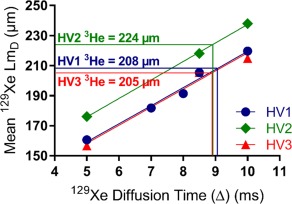
Mean ^129^Xe Lm_D_ results at different ^129^Xe diffusion times for three healthy volunteers. A strong linear dependence in ^129^Xe diffusion time and mean ^129^Xe Lm_D_ value was obtained for HV1 (*r* = 0.98, *P* = 0.015). When the Δ = 8.5 ms results for HV1 was considered, the diffusion time Δ = 9.1 ms corresponded to the subject's ^3^He Lm_D_ value. A similar diffusion time trend was observed in the other two healthy volunteers (HV2 and HV3).

Mean ^129^Xe ADC values (at b = 12 s/cm^2^) decreased nonlinearly with increasing diffusion time; a trend observed previously in ^3^He ADC measurements 4, 30. The logarithmic relationship observed between ^129^Xe ADC and diffusion time also matches the trend observed for ^3^He ADC 30. The SEM‐derived Lm_D_ values exhibited a strong positive linear dependence with Δ over the range of 5–10 ms. The dependence of Lm_D_ on Δ reflects the changes in the theoretical characteristic free diffusion lengths probed for each experiment. At Δ = 10 ms, corresponding to the characteristic free diffusion length of ^129^Xe ( $\sqrt{2D_{0}\Delta }$  = 530 µm) which is identical to the free diffusion length of ^3^He in air for the diffusion times used by Chan et al. 10, a mismatch of Lm_D_ values was still observed in the data from three healthy volunteers (Fig. 7).

This mismatch suggests that even at the same characteristic free diffusion length there may be inherent differences in the specific diffusion dephasing regime of the respective gas in the lung alveoli which makes this assumption of Gaussian relation between diffusion length and diffusion time inexact. The differences in diffusion dephasing regime stems from intrinsic properties (i.e., gyromagnetic ratio and diffusivity) of each gas, and thus leads to different mechanisms that contribute to non‐Gaussian diffusion signal behaviors that are not accounted for in the calculation of characteristic free diffusion length. For example, differences in the diffusional dephasing regime due to microscopic background susceptibility gradients may exist between ^129^Xe and ^3^He at the same field strength due to the smaller gyromagnetic ratio of ^129^Xe. These effects on diffusive length scales are similar to the effect of different B_0_ field strengths on ^3^He ADC values 5.

### Benchmarking of Empirically Optimized ^129^Xe Diffusion Time

The decision to further accelerate with four‐fold undersampling was motivated by the need to reduce the breath‐hold duration incurred with ^129^Xe diffusion times > 5 ms. To verify that good agreement in Lm_D_ values was obtained with three‐ and four‐fold undersampling, all five healthy volunteers were imaged with an additional AF = 3 ^129^Xe CS acquisition at ^129^Xe Δ = 8.5 ms. A slice‐by‐slice comparison of mean Lm_D_ values for the five healthy volunteers was performed, and Bland‐Altman analysis confirmed a mean bias of +1.5% (+2.9 µm) for AF = 4. The 95% confidence interval of −6.9% to + 10.0% (−13.4 to 19.3 µm) was within typical standard deviation values of lung Lm_D_ values in healthy volunteers. This slight increase in mean slice Lm_D_ values obtained with AF = 4 is likely the result of CS reconstruction error associated with increased undersampling. In addition, the broad 95% confidence interval range could also be explained by inexact coregistration of image slices due to slight changes in subject position between the AF = 3 and AF = 4 scan sessions. However, the small increase in Lm_D_ justifies that implementation of AF = 4 in prospective acquisitions with ^129^Xe Δ = 8.5 ms. The reduction of scan time to within 16 s is more tolerable for a wider range of subjects, therefore AF = 4 will be used in all subsequent 3D multiple b‐value ^129^Xe DW‐MRI acquisitions.

Using the empirically optimized diffusion time, ^129^Xe‐derived Lm_D_ values demonstrated improved matching with ^3^He Lm_D_ at ^129^Xe Δ = 8.5 ms than at ^129^Xe Δ = 5 ms. The mean difference between whole lung ^129^Xe and ^3^He Lm_D_ values across all subjects was −2.2%, and the mean bias in individual lung ^129^Xe Lm_D_ values was −2.1%. ^129^Xe Δ = 8.5 ms was derived from preliminary data, and this small bias may suggest that a different optimal diffusion time (slightly longer than Δ = 8.5 ms) could be used to bring the bias toward 0%. Considering Δ = 8.5 ms Lm_D_ for HV1, a ^129^Xe Δ = 9.1 ms was found to match the volunteer's ^3^He Lm_D_ value (Fig. 7). Additionally, when the previous ^129^Xe Δ = 5 and 8.5 ms results for HV2 and HV3 are considered in conjunction with an additional acquisition at ^129^Xe Δ = 10 ms, a similar optimal diffusion time of around 9 ms was obtained as well (Fig. 7). Nevertheless, the observed bias of −2.1% is equivalent to the same‐day reproducibility error (2.1%) 31 of Lm values calculated from multiple b‐value ^3^He DW‐MRI using the CM. This indicates that any mismatch between ^3^He and ^129^Xe Lm_D_ values at the ^129^Xe Δ = 8.5 ms is of the order of same‐day reproducibility error, and we conclude that comparable lung morphometry maps can be obtained with ^129^Xe.

One limitation of this study is that the ^129^Xe diffusion time was optimized based upon the Lm_D_ results from healthy volunteers only. In subjects with emphysematous changes to alveolar length scales, a different relationship between ^129^Xe Lm_D_ and diffusion time may exist. However, the strong agreement between ^129^Xe and ^3^He Lm_D_ results from the subsequent prospective acquisitions in healthy volunteers, ex‐smokers, and COPD patients suggests that ^129^Xe Δ = 8.5–9 ms is valid across a range of alveolar sizes subject to age and smoking‐related emphysema.

The empirically optimized ^129^Xe Δ = 8.5 ms used in our study is significantly longer than the diffusion time used in other ^129^Xe lung morphometry studies. In Sukstanskii and Yablonskiy 18, ^129^Xe Δ = 5 ms was chosen and CM phenomenological expressions for acinar airway geometrical parameters were also recalibrated for ^129^Xe such that lung morphometry results matched those of ^3^He. However, it was noted that if the same theoretical free diffusion length is probed with both nuclei, the ^3^He‐based phenomenological expressions can be applied to derive ^129^Xe lung morphometry parameters 18. In a small subset of the preliminary study cohort (HV1–HV4), the assumption that, like the SEM, the CM will yield more comparable lung morphometry results at ^129^Xe Δ = 8.5 ms than with ^129^Xe Δ = 10 ms was explored. ^129^Xe Δ = 8.5 and 10 ms data were analyzed with ^3^He‐based CM parameters, and derived Lm was compared with ^3^He‐derived Lm values. A mean difference of 4.3% was obtained between ^129^Xe Δ = 8.5 ms Lm and ^3^He Lm, whereas at ^129^Xe Δ = 10 ms the difference was larger (11.5%). These results, albeit in a small subset of subjects, support the implementation of the ^3^He‐based CM with ^129^Xe DW‐MRI at ^129^Xe Δ = 8.5 ms.

The mean ^3^He Lm values for healthy volunteers (∼180 µm), ex‐smokers (∼250 µm), and COPD patients (∼500 µm) were consistent with previously reported ^3^He Lm values 7, 32, 33. The mean ^129^Xe Lm for ex‐smokers (with ^129^Xe Δ = 8.5 ms) are also in agreement with previous ^129^Xe Lm values reported at 3 T obtained with ^129^Xe Δ = 5 ms 20, 21. The ^129^Xe Lm for the GOLD II COPD subject (318 µm) is also comparable to the ^129^Xe Lm (∼350 µm) reported in COPD patients (GOLD I‐III) 20, 21. When ^129^Xe Lm from the ^129^Xe Δ = 8.5 ms data was evaluated with ^3^He‐based CM, an overall mean difference of +1.1% and +2.3% was obtained for whole lung and individual lung ^129^Xe and ^3^He Lm values, respectively. This small bias is of a similar magnitude as that observed with SEM‐derived Lm_D_ and therefore suggests that ^129^Xe lung morphometry results obtained with ^129^Xe Δ = 8.5 ms are comparable to ^3^He results analyzed with both the cylinder and stretched exponential models.

## Conclusions

With limited availability of ^3^He, there is a strong motivation to evaluate functional and structural information that can be derived from the readily available and cheaper ^129^Xe gas isotope. Compressed sensing has facilitated acquisition of single‐breath 3D multiple b‐value ^129^Xe DW‐MRI for whole lung morphometry mapping. SEM‐derived Lm_D_ demonstrated a linear dependence with diffusion time, and the best agreement between ^129^Xe and ^3^He Lm_D_ results was obtained with an empirically optimized ^129^Xe Δ = 8.5 ms. Prospective CS acquisitions were used to validate ^129^Xe Δ = 8.5 ms in healthy volunteers, ex‐smokers, and COPD patients, and a strong agreement (mean Lm_D_ bias of −2.2%) in ^129^Xe and ^3^He Lm_D_ values was obtained. A similar level of agreement (mean Lm bias of +1.1%) was obtained with CM‐derived Lm, indicating that ^129^Xe DW‐MRI acquired with ^129^Xe Δ = 8.5 ms is a viable alternative to ^3^He for 3D whole lung morphometry assessment with both cylinder and stretched exponential models.
